# Effective treatment of gallbladder neuroendocrine carcinoma with nivolumab

**DOI:** 10.1002/ccr3.8040

**Published:** 2023-10-11

**Authors:** Shinichiro Kanda, Kazuhiro Hiyama, Izumi Kirino, Yasuo Fukui, Hideo Terashima

**Affiliations:** ^1^ Kochi Medical School (KMS) Kochi University Kochi Japan; ^2^ Department of Surgery Atagi Hospital Kochi Japan

**Keywords:** EUS‐FNA, gallbladder NEC, immune checkpoint inhibitors

## Abstract

An 89‐year‐old patient with gallbladder neuroendocrine neoplasms (GB‐NENs) and lung cancer metastasis underwent nivolumab monotherapy, resulting in tumor shrinkage. Surgery and adjuvant nivolumab showed efficiency despite low expression of PD‐L1.

## Graphical abstract

During the follow‐up PET‐CT for pulmonary adenocarcinoma, accumulation was detected around the gallbladder. EUS‐FNA confirmed the presence of small‐cell neuroendocrine carcinomas (SCNECs). Chemotherapy proved intolerable, leading to the initiation of nivolumab monotherapy, resulting in tumor shrinkage. Subsequent surgery and adjuvant nivolumab yielded positive outcomes. The postoperative pathology diagnosed the gallbladder tumor as NECs, with an extremely low PD‐L1 expression. Nivolumab may be effective in GB‐NEN despite low PD‐L1 expression.

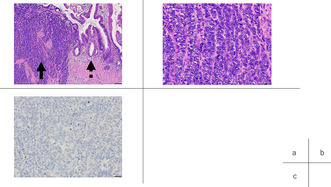

## 1 INTRODUCTION

Neuroendocrine neoplasms (NENs) often occur in the pancreas (P‐NENs) and gastrointestinal tract (GINENs).^1^ Gallbladder (GB) NENs are rare and account for only 0.5% of all NENs and 2% of all gallbladder tumors.^2^ Diagnosing GB‐NENs before surgery is challenging due to a lack of specific manifestations, and to radiological findings that resemble those of other GB tumors. Most GB‐NENs are detected incidentally during histological examination of GB samples after surgical removal, often performed during cholecystectomy for cholecystitis, or for suspected biliary malignancy.^3^, ^4^, ^5^, ^6^, ^7^, ^8^

Although there is no consensus on the treatment of gallbladder neuroendocrine tumors, early in situ GB‐NENs typically respond favorably to cholecystectomy alone, whereas late‐stage tumors require radical surgeries involving lymph node dissection and potential removal of nearby metastatic lesions.^9^, ^10^ Currently, there are no diagnostic imaging findings for GB‐NENs. Platinum‐based therapeutants are generally recommended for GB‐NENs, however, their efficacy is unfavorable.^11^ Furthermore, patients with GB‐NENs have poor prognosis.^12^ To date, there has been only one report suggesting effectiveness of nivolumab.^13^ Here, we report a case involving successful treatment of a GB‐NEN with nivolumab.

## 2 CASE HISTORY/EXAMINATION

An 89‐year‐old Japanese man presented to our department with gallbladder cancer. He had no disability nor any signs of cognitive dysfunction.

**FIGURE 1:**
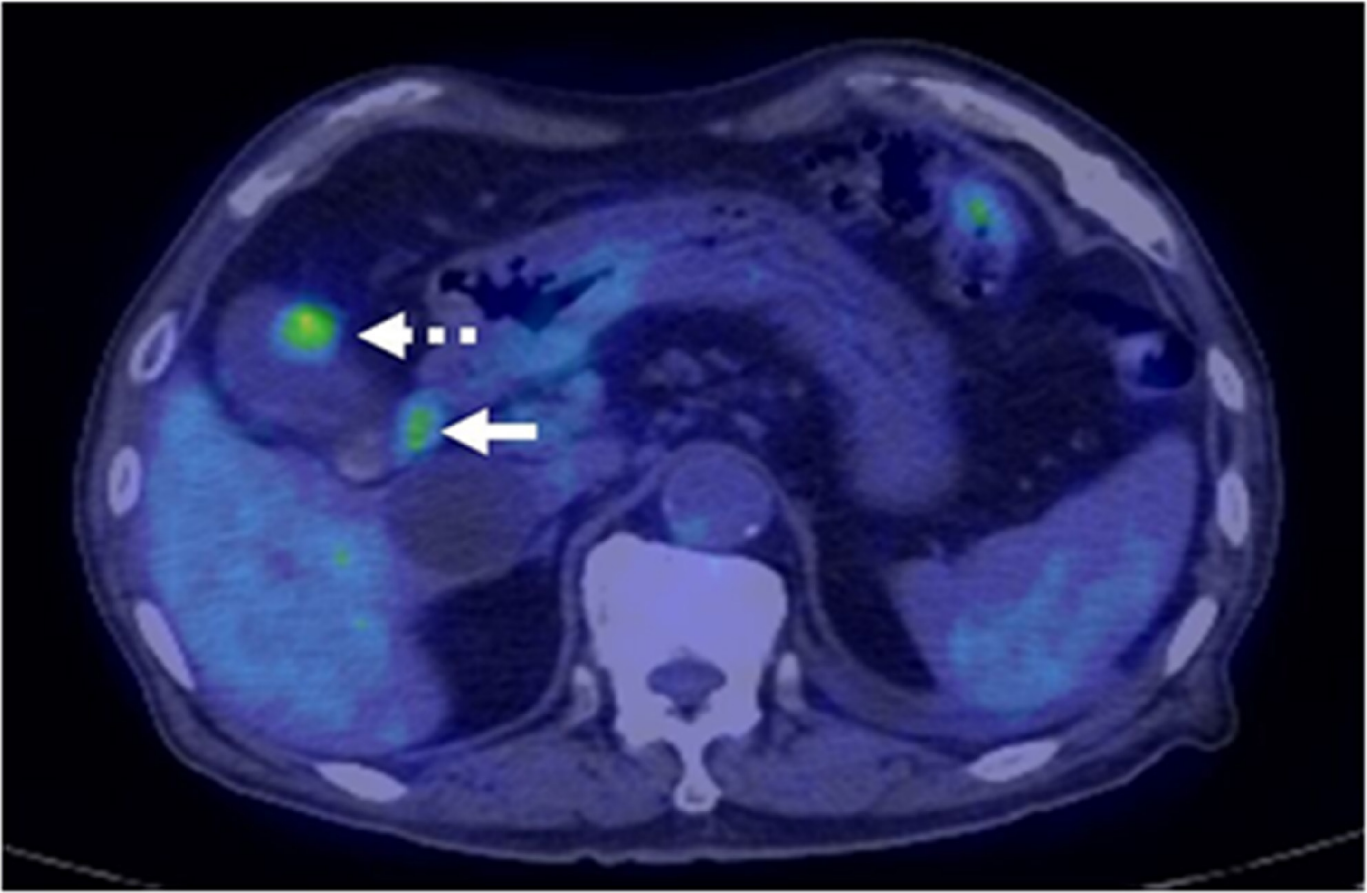
PET‐CT revealed FDG accumulated in the fundus of the gallbladder (SUV max:4.2) (dotted arrow) and hepatoduodenal lymph nodes (SUVmax:3.2) (solid arrow) as well as lymph nodes of the mediastinum.

He had been suffering from left upper lobe (S1+2) lung cancer (papillary adenocarcinoma, pT3N2M0, pStage IIIB). Left upper lobectomy with regional and left mediastinal lymphadenectomy had been performed 3 years prior, but was followed by mediastinal lymph node metastases and an intrapulmonary metastasis in the right lobe 2 years before. The patient received chemoradiotherapy which stabilized the metastases. The patient's medical comorbidities included left internal carotid artery stenosis, early colorectal cancer, early bladder cancer, hypertension, and hyperuricemia. The patient had no abdominal symptoms or physical examination findings, but follow‐up PET‐CT revealed that^18^ F‐fluorodeoxyglucose (FDG) had accumulated in the fundus of the gallbladder (Standard Uptake Value (SUV) max:4.2) and the hepatoduodenal lymph nodes (SUVmax:3.2), as well as in the mediastinum lymph nodes (right carina lymph node SUVmax:11.0, para‐aorta lymph node SUVmax:6.1). A tumor in the gallbladder and those in the hepatoduodenal lymph nodes had diameters of 15 and 12 mm, respectively. No significant change was observed in lung tumor size (Figure 1).

## 3 DIFFERENTIAL DIAGNOSIS, INVESTIGATION, AND TREATMENT

As a result of the examination, the patient was diagnosed with primary GB cancer and metastatic lung cancer of the right lower lobe (S6) (adenocarcinoma, cT1N2 M0, cStage IIIA). Endoscopic ultrasound‐guided fine needle aspiration (EUS‐FNA) of lymph nodes in the hepatoduodenal ligament revealed small‐cell neuroendocrine carcinomas (SCNECs), which indicated primary GB cancer with lymph node metastasis, rather than metastases of the lung adenocarcinoma.

**FIGURE 2:**
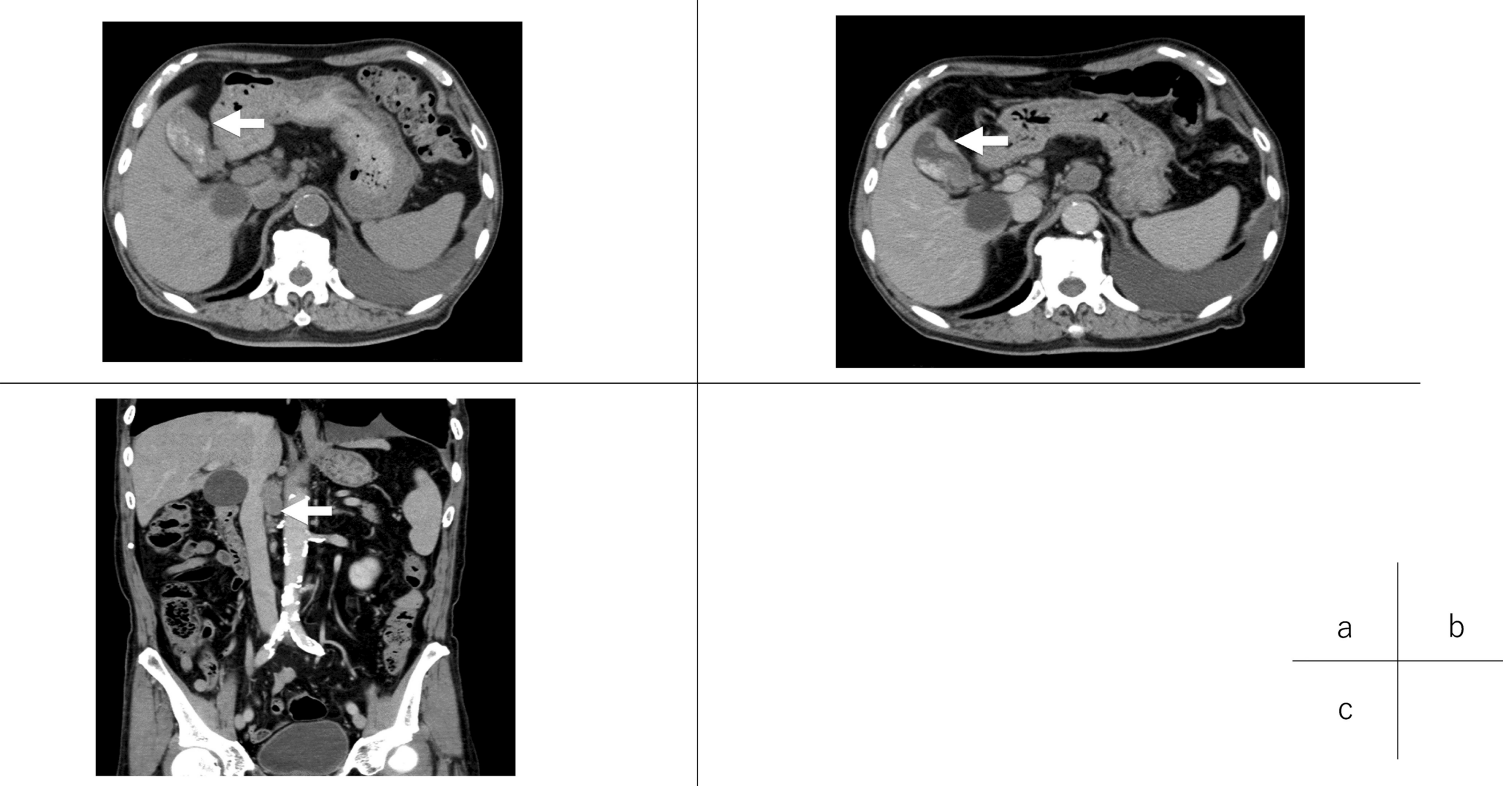
CT images (A) before chemotherapy and (B, C) after 10 courses of nivolumab. After nivolumab, the tumor had clearly shrunk (B, arrow), but limited lymphadenopathy proximal to the abdominal aorta emerged (c, arrow).

**FIGURE 3:**
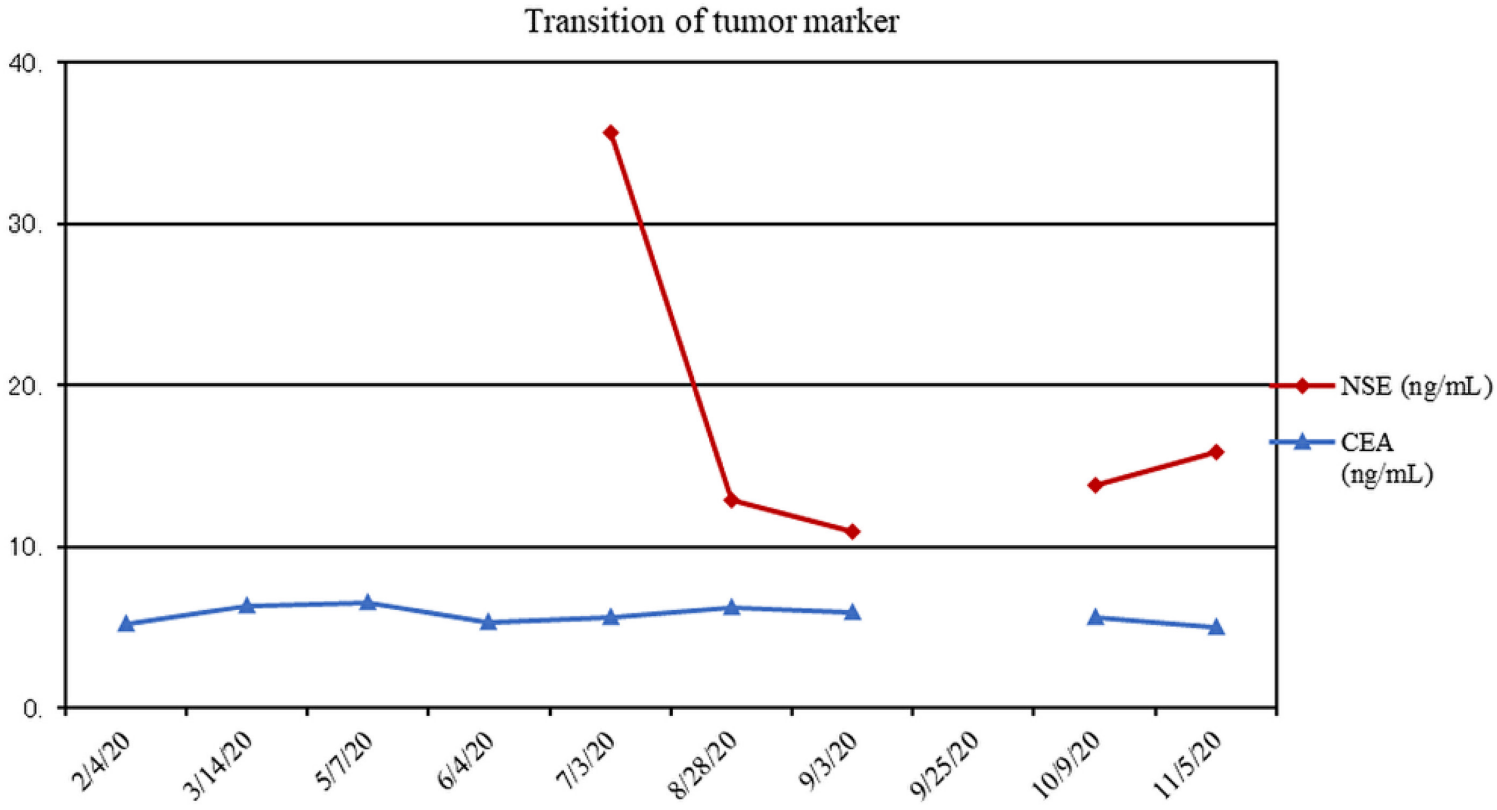
NSE turned negative after 10 courses of nivolumab.

Generally, prognosis of GB carcinoma with lymph node metastasis and SCNEC is quite poor.^14^, ^15^ The patient, an octogenarian, had performance status of 0 (CTC Version 2.0), but he had various critical comorbid diseases such as recurrent lung cancer. Therefore, we first recommended chemotherapy instead of definitive surgery. However, the standard platinum‐based regimen of SCNEC proved intolerable because of his general status. Although there are few studies suggesting that nivolumab is effective against neuroendocrine carcinoma and GB cancers,^13^, ^16^ nivolumab monotherapy (240 mg, every 2 weeks) was initiated. At that time, neuron‐specific enolase (NSE) was significant (35.7 ng/mL). After 10 courses of nivolumab, a CT scan showed that the GB tumor had shrunk without remarkable lymphadenopathy in the hepatoduodenal ligament, but with limited lymphadenopathy adjacent to the abdominal aorta (Figure 2). A tumor marker, NSE, turned negative (12.9 ng/mL) (Figure 3). Even though prognosis of GB carcinoma with lymph node metastases is quite poor (median OS: 13.5 months),^17^ radical resection of the tumor can prolong survival in cases in which metastases disappear after chemotherapy. Therefore, we decided to operate, for the following reasons: (1) We could remove the tumor completely with lymphadenectomy next to the abdominal aorta. (2) If the tumor had increased during further nivolumab monotherapy, we would have been unable to achieve complete surgical resection.

Furthermore, surgery seemed likely to be tolerated by the patient because he was fairly healthy and because the surgery did not require major hepatectomy or bile duct resection. We carefully explained the foregoing to the patient and his family and obtained consent for surgery. Then, an extended cholecystectomy and lymphadenectomy around the hepatoduodenal ligament and sampling of lymph nodes proximal to the abdominal aorta were performed.

## 4 OUTCOME AND FOLLOW‐UP

**FIGURE 4:**
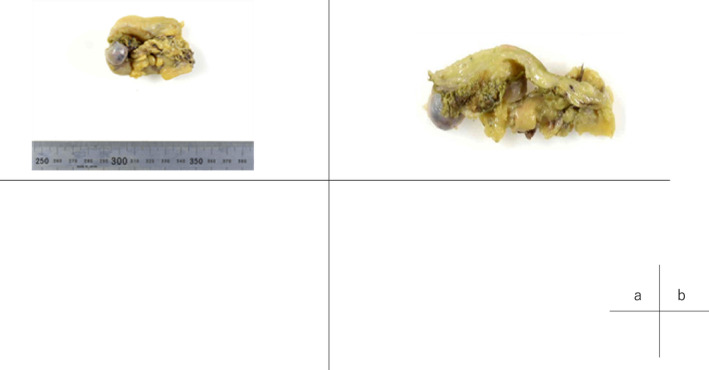
There was a mass (15 × 8 × 10 mm) on the lower side of the gallbladder, which was atrophied, with a thickened wall overall.

**FIGURE 5:**
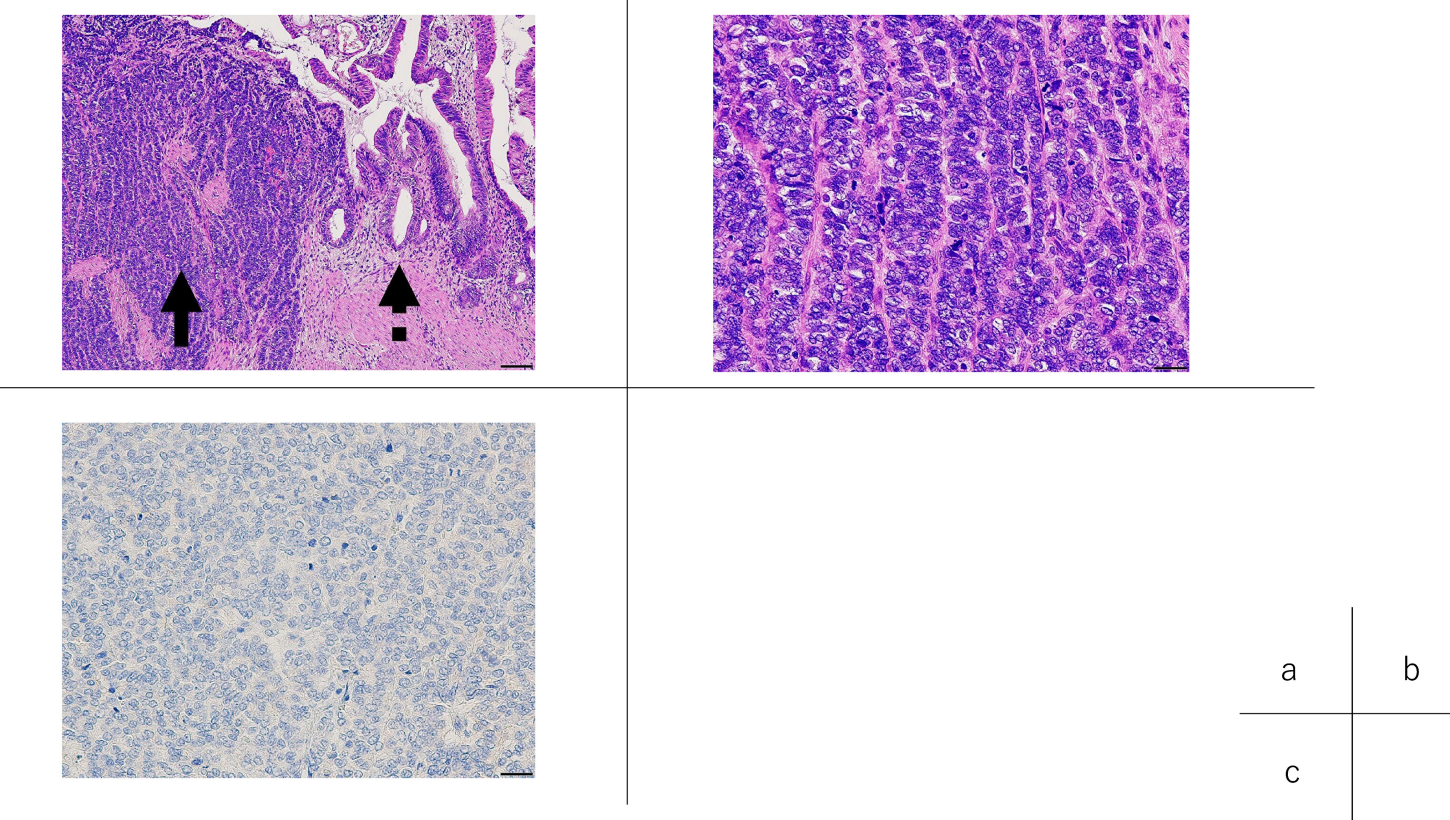
NEC (solid arrow) and adenocarcinoma (in situ) components (dotted arrow) were mixed (A). In the NEC component, the N/C ratio was increased, and abnormal mitosis was observed (B). Furthermore, glandular duct structure disappeared, and solid growth of NEC was observed (B). PD‐L1 expression is rarely seen (C).

After surgery, no major adverse events were observed, except delayed gastric emptying, Clavien dindo grade II, and acute gastric mucosal lesions, Clavien dindo grade IIIa. Histopathologically, the atrophied gallbladder had a tumor in its fundus (15 × 8 × 10 mm). (Figure 4). Upon microscopic examination, NEC and adenocarcinoma (in situ) components were mixed (Figure 5). Most components of the tumor were NEC, and adenocarcinoma in situ (AIS) was present at both ends of the NEC. Histologically, there was no metastasis in the hepatoduodenal ligament, but there was metastasis beside the abdominal aorta. PD‐L1 28‐8 IHC showed that the PD‐L1 expression rate was less than 10% (Figure 5). Histological evaluation of the chemotherapeutic effect was Grade 0, no change (6th Ed. of General Rules for Clinical and Pathological Studies of Cancer of the Biliary Tract). One month after surgery, adjuvant nivolumab monotherapy was initiated. Three months have elapsed as the surgery, and no recurrent lesion has been observed.

## 5 DISCUSSION

Primary GB‐NENs account for 0.5% of all NENs and 2.1% of all GB cancers.^18^ The most common primary tumor sites are the gastrointestinal and respiratory tracts,^2^ and GB‐NECs are very rare. Although there is no comprehensive epidemiological information on gallbladder NEC, there is a report that the median age of patients is 58.4 years (range 26–75), with an M:F ratio of 7:8. There are no symptoms specific to GB‐NENs. The most common symptom is right upper quadrant pain, which may be accompanied by nausea, vomiting, and a positive Murphy's sign.^10^, ^19^, ^20^, ^21^, ^22^, ^23^ Some patients also present with weight loss and anorexia.^23^ There were no subjective symptoms or significant physical findings in this patient. Median overall patient survival is 26 months for those without lymph node metastasis and 10.4 months for patients with it.^24^ Cisplatin and Etoposide (EP therapy) or cisplatin and irinotecan (IP therapy) is widely recommended for NEC, but the response rate and median overall survival (OS) are unfavorable (EP: response rate, 12%; median OS 6.9 months, IP: response rate, 39%; median OS 10.1 months).^11^

In the present case, considering the patient's age and medical history, we started nivolumab monotherapy. As a result of image examination, though the paraaortic lymph node swelled, the GB tumor clearly shrank, and swelling of hepatic duct lymph node disappeared. A tumor marker against NEC, NSE turned negative.

These findings suggest that nivolumab can be effective against GB‐NEC. Nevertheless, PD‐L1 28‐8 immunohistochemistry (IHC) was positive in less than 10% of cancer cells. This suggests the following two possibilities: (1) PD‐L1 positive cells were killed by nivolumab, leaving only colonies of negative cells. (2) Nivolumab activates T cells, which kill PD‐L1 negative cells. Drug sensitivity of cancer cells varies from cell to cell because each cancer cell has a different genetic background. Therefore, although ICIs are very effective for cancer cells with high PD‐L1, ICIs may also be effective against subgroups with low or undetectable PD‐L1. Actually, it has been reported that in lung cancer, ICIs respond even when PD‐L1 expression is extremely low.^25^ Recent studies suggest that PD‐L1 inhibitors themselves may activate tumor‐reactive T cells and enhance anti‐tumor immunity.^26^ Further studies on the correlation between the PD‐L1 expression rate and the ICI response rate in gallbladder cancer are awaited.

EUS‐FNA is quite useful because GB cancer sensitivity is 96%.^27^ In this case, we detected NEC using EUS‐FNA for lymph nodes in the hepatoduodenal ligament. However, in cases in which primary tumors have both NEN and nonneuroendocrine components, so‐called mixed neuroendocrine‐nonneuroendocrine neoplasms (MiNENs), EUS‐FNA may not be able to detect all of them. NEC is highly malignant and readily metastasizes to other tissues. Therefore, even if an NEC component is detected by biopsy of metastatic lymph nodes, the primary tumor may contain nonneuroendocrine components such as adenocarcinoma.

It is necessary to choose a chemotherapy regimen that is effective for all tumor components. Multiple EUS‐FNA enables collection of multiple samples, which can facilitate correct diagnosis and selection of an appropriate chemotherapy regimen, but dissemination and bile leakage are problematic.^28^ Furthermore, NEC is located deep in an area of vascular or perineural invasion.^29^ As a result, most MiNEN are diagnosed from surgical specimens.^30^ Therefore, it is important to diagnose using serum tumor markers, imaging tests, or EUS‐FNA. Ultimately, it may be useful to collect samples surgically.

In conclusion, EUS‐FNA is useful for diagnosis of GB‐NECs. However, because the primary tumor may be MiNEN rather than NEC, it is better to perform a biopsy when selecting a chemotherapy regimen. Nivolumab may enhance the immune function of T cells by some means other than inhibition of PD‐1, and it may be effective against GB‐cancers involving NEC despite the low expression of PD‐L1.

## AUTHOR CONTRIBUTIONS

**Shinichiro Kanda:** Writing – original draft. **Kazuhiro Hiyama:** Supervision. **Izumi Kirino:** Supervision. **Yasuo Fukui:** Supervision. **Hideo Terashima:** Supervision.

## FUNDING INFORMATION

None.

## CONFLICT OF INTEREST STATEMENT

The authors declare that they have no conflicts of interest and that there are no relevant financial disclosures to report.

## CONSENT

Written, informed consent was obtained from the patient for publication of this case report and accompanying images. A copy of the written consent is available for review upon request.

## Data Availability

All data underlying the results are available as part of the article and no additional source data are required.
